# Persistence of positive skin test 25 years after a penicillin-induced anaphylaxis

**DOI:** 10.1186/1939-4551-8-S1-A87

**Published:** 2015-04-08

**Authors:** Ana Carolina D'onofrio-Silva, Eduardo Longen, Marcelo Vivolo Aun, Marisa Rosimeire Ribeiro, Laila Sabino Garro, Nathália Coelho Portilho, Jorge Kalil, Pedro Giavina-Bianchi, Antonio Abílio Motta

**Affiliations:** 1 University of São Paulo, Brazil

## Background

Skin tests are important in the investigation of hypersensitivity drug reactions (HDR), particularly when betalactams are involved. However, the sensitivity decreases with time. It has been described that skin tests become negative about five years after a betalactam hypersensitivity reaction. We report a patient with persistence of a positive skin test twenty five years after an anaphylactic reaction due to penicillin.

## Methods

Literature review and case description.

## Results

We assessed a 39 years old female with a history of severe penicillin allergic reactions in the past. She reported two reactions after taking benzathine benzylpenicillin intramuscularly (IM) in 1989. Firstly, thirty minutes after she took a dose because of a urinary tract infection, she developed urticaria on her arms. She was treated with antihistamines and told to take a second dose after a week. When she took the second dose, she developed a severe reaction compatible to anaphylaxis: urticaria, angioedema, bronchospasm and asfixia. She was not treated with epinephrin, but she got better after taking corticosteroids, antihistamines and bronchodilators. She was then told to avoid betalactams in the future. She had never taken those drugs again, but she used to have contact urticaria when preparing amoxicillin to their children. In 2014, she was diagnosed as having syphilis in pregnancy. Then, she was referred to our outpatient clinic specialized in HDR so that we could perform a desensitization to benzathine benzylpenicillin. First of all we performed skin tests with penicillin G potassium 10.000UI/mL. The prick test was negative, but the intradermal one was positive, confirming the presence of specific IgE. During the desensitization, she develop palm and sole pruritus and rhinoconjuncitivitis, but the procedure was concluded successfully and she could be treated with the whole dose of 7.200.000UI by IM route.

## Conclusions

Despite the literature data, we reported a patient having a positive skin test to penicillin a long time after the initial immediate reaction. We speculate that she continued being stimulated by cutaneous contact with amoxicillin, maintaining specific IgE production. In conclusion, skin tests should always be performed before a challenge procedure, specially when the initial reaction was severe.

## Consent

Written informed consent was obtained from the patient for publication of this abstract and any accompanying images. A copy of the written consent is available for review by the Editor of this journal.

